# Dynamic regulation of plasmodesmatal permeability and its application to horticultural research

**DOI:** 10.1038/s41438-019-0129-3

**Published:** 2019-04-06

**Authors:** Yanbiao Sun, Dingquan Huang, Xu Chen

**Affiliations:** 1 College of Life Science and Fujian Provincial Key Laboratory of Haixia Applied Plant Systems Biology, Fujian Agriculture and Forestry University, Fuzhou, Fujian China; 2 Haixia Institute of Science and Technology, Horticultural Plant Biology and Metabolomics Center, Fujian Agriculture and Forestry University, Fuzhou, 350002 China

**Keywords:** Molecular engineering in plants, Molecular engineering in plants, Molecular engineering in plants, Molecular engineering in plants

## Abstract

Effective cell-to-cell communication allows plants to fine-tune their developmental processes in accordance with the prevailing environmental stimuli. Plasmodesmata (PD) are intercellular channels that span the plant cell wall and serve as cytoplasmic bridges to facilitate efficient exchange of signaling molecules between neighboring cells. The identification of PD-associated proteins and the subsequent elucidation of the regulation of PD structure have provided vital insights into the role of PD architecture in enforcing crucial cellular processes, including callose deposition, ER–Golgi-based secretion, cytoskeleton dynamics, membrane lipid raft organization, chloroplast metabolism, and cell wall formation. In this review, we summarize the emerging discoveries from recent studies that elucidated the regulatory mechanisms involved in PD biogenesis and the dynamics of PD opening-closure. Retrospectively, PD-mediated cell-to-cell communication has been implicated in diverse cellular and physiological processes that are fundamental for the development of horticultural plants. The potential application of PD biotechnological engineering represents a powerful approach for improving agronomic traits in horticultural crops in the future.

## Intercellular channels: managing communications

Modifying the structure and dynamics of plasmodesmata (PD), channels that directly connect the cytoplasm of neighboring plant cells, could improve the growth and productivity of horticultural crops. Xu Chen and colleagues at Fujian Agriculture and Forestry University, China, review recent understanding of the mechanisms regulating the opening and closing of PD and potential applications of this knowledge. Changes in the levels of the sugar polymer callose or phospholipids lining the membrane of PD, or disrupted delivery and transport of PD-associated proteins affect the channels’ permeability. Modulating PD dynamics could not only enhance plant survival, by preventing the spread of pathogens and improving their response to changing environmental conditions, but also influence crop yield by aiding the transport of nutrients into fruits and the establishment beneficial symbioses.

## Introduction

Plasmodesmata (PD) are membrane-lined channels that transverse the plant cell wall and function as conduits to allow the exchange of various cellular molecules between plant cells^1^. The architecture of PD is diverse even within a single plant species and can be classified into simple, branched (Y-shaped) or twinned (X-shaped) forms based on plant developmental stage and tissue speciticity^2,3^. PD morphology also reflects cell function and correlates explicitly with the intercellular transportation requirements of individual cells. With the help of transmission electron microscopy (TEM), the PD ultrastructure has been determined^4^. According to these observations, the center portion of PD consists of cylindrically compressed endoplasmic reticulum (ER), known as the desmotubule^2,5^. The gap between the desmotubule and the plasma membrane (PM) constitutes the major conduit (cytoplasmic sleeve) to facilitate the passage of molecules. The desmotubule and the inner leaflet of the PD membrane are connected by spoke-like elements, which are hypothetically actin and myosin-related components of the cytoskeleton^6^. PD membranes contain both membranes from the PM and ER^4^. The apposition between these two membranes constitutes a highly specialized type of membrane contact site. A previous report suggested that a very tight connection occurs at the membrane contact site during the early stage of PD biogenesis^4^. On these juxtaposed PD membranes, specialized membrane nanodomains are enriched with specific sets of lipids and protein constituents, which are crucial for controlling the flexibility of the PD membrane^7^.

In the current model, the cytoplasmic sleeve, which is defined as the space available for molecular trafficking, imposes a size exclusion limit on PD permeability^4^. The deposition of callose (a polysaccharide molecule) at the peripheral neck region of the PD regulates the plasticity of the PD orifice in a highly controlled manner^8^. Although details regarding PD architecture are well-established, PD biogenesis and the regulatory mechanisms of PD conductivity are still unclear. Therefore, in-depth analyses of PD components and their impact on intercellular permeability will be critical to provide further insights into the regulatory mechanisms of PD. In this article, we discuss recent research progress with regard to the regulatory mechanisms of PD permeability and dynamics. We also provide new insights into the applications of PD bioengineering techniques in promoting the growth and productivity of horticultural crops.

## Regulators that control the PD aperture

**Fig. 1:**
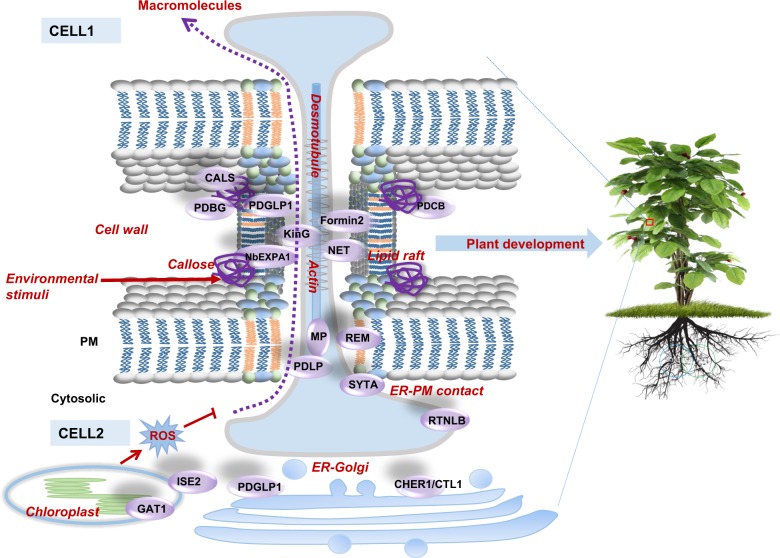
Dynamic regulation of PD conductivity for plant development. Dynamic PD opening-closure is controlled by the intracellular components of callose, PM-lipid raft, actin, ER–desmotubule, ER–Golgi, ER–PM contact, cell wall, and chloroplast. PD-associated proteins that are individually involved in distinct processes of PD regulation are summarized in the left panel (shown as purple balls). Environmental stresses also influence PD conductivity through the regulation of callose homeostasis

To elucidate the regulatory machinery of PD conductivity, Fernandez-Calvino et al.^9^ isolated 1341 putative PD-associated proteins from *Arabidopsis* suspension cultured cells, including known plasmodesmal proteins, e.g., plasmodesmata-located proteins (PDLPs), β-1,3-glucanase (BGs), calreticulin, and remorin (REM), as well as the putative PD-associated regulators, e.g., glycosylphosphatidylinositol (GPI)-anchored proteins, receptor-like kinases, and transmembrane proteins. Although this extraction approach led to contamination with some residual cytoplasmic proteins, the PD-proteome data nevertheless provide a valuable resource for further elucidation of the functionality of PD and signaling transduction that occurs at the PD^9^. A subsequent study by Grison et al. adopted a more rigorous isolation approach and identified 41 PD-abundant proteins, including plasmodesmata callose binding proteins (PDCBs), BGs, callose synthases (CalS), and tetraspanins, strengthening the evidence that PD-enriched membrane fractions display a distinct protein profile compared with the neighboring PM^7^. With the help of these PD-proteome data, complementary validations by genetics and protein function studies, we now have numerous exciting discoveries that help us to understand the regulatory mechanisms of PD architecture/functionality (Fig. 1).

### Callose turnover

The β*-*1,3-glucan polymer callose is deposited within the neck regions of PD. The turnover of callose is highly regulated through synthesis by CalS and specific PD-localized BGs, which reduce callose deposition^8^. Numerous studies have shown that callose participates in fine-tuning the opening/closure dynamics of the PD, as shown by the fact that excessive callose deposition decreases the PD aperture and a reduction in callose increases PD permeability (see ref. ^8,10,11^ for recent reviews). Vatén et al.^12^ devised an inducible synthetic allele of the CalS3 (*icals3m*) system to manipulate callose deposition during plant development. This system has been employed to block the movement of non-cell autonomous proteins (e.g., SHORT-ROOT (SHR) protein) via PD and controls cell division, cell expansion and cell polarity formation during root growth^13,14^. This tool has been widely used to elucidate the regulatory networks of tissue-specific symplastic networks and the function of symplastic communication in plant development^13–18^.

A common acute response of plants to biotic or abiotic stress is an alteration of callose-mediated PD permeability with the accompanying regulation of cell-to-cell signaling^19,20^. The available evidence shows that excess copper/iron induces PD callose deposition, thereby decreasing the size of the PD aperture mechanically^20^. The limited PD permeability subsequently blocks the uptake of metal to ensure the survival of plants in the presence of metal toxicity^20^. Meanwhile, the accumulation of callose caused by metal toxicity promotes cellular integrity through the facilitation of frequent interactions between cell wall components^20^. For example, in phosphate-starved root tips, iron and callose are both deposited in the apoplast of the stem cell niche of the root apical meristem and thus inhibit PD-dependent symplastic communication^21^. Interestingly, iron accumulation and phosphate deficiency coincide at the site of callose deposition, implying that phosphate and iron availability affect the PD structure in stressed environmental conditions^21^. Thus, callose is considered as a significant component that regulates the structure and functional dynamics of PD at different plant growth stages and under diverse biotic/abiotic stresses (see ref. ^8,10,11^ reviews).

### ER–Golgi secretory pathway

Given the importance of PD in the cell-to-cell transport of ions, researchers have identified the choline transporter-like gene (*CTL1*) as a new regulatory component that influences the development of PD^22^. CTL1 localizes to the *trans*-Golgi network (TGN). Deficiency of CTL1 causes the mislocalization of PDCB1 to the intracellular TGN compartment and impairs PD morphology^22^. The intracellular retention of PDCB1 in the *significant ionome changes 1* (*sic1)* mutant (a mutant of the CTL1 gene) indicates that the subcellular distribution of PDCB1-labeled PD requires the ER–Golgi-mediated secretory pathway. CTL1 is involved in choline homeostasis and lipid metabolism, and choline sequestration into vesicles facilitates the asymmetric distribution of phospholipids in the vesicle membrane and PM. Correspondingly, a steady reduction of phospholipids in the *sic1* mutant is an indication that CTL1 mutation likely causes shrinkage of the cellular membranes. Thus, PD defects exhibited by the *sic1* mutant are the result of changes in the level of membrane phospholipids^22^. These observations adequately underscore the importance of lipids and the ER–Golgi secretory pathway in the development of PD.

The trafficking and accumulation of PD-localized proteins (called PDLP1) is also mediated by the ER–Golgi secretory pathway. Inhibition of the ER–Golgi secretory pathway results in the retention of PDLP1 at the ER and abolishes the PD localization pattern of PDLP1^23^. More importantly, PDLP family proteins provide an anchoring signal for the recognition of virus movement proteins (MPs)^24^. Following virus infection, PDLPs interact with MPs to promote the assembly of MPs into tubules within the PD and facilitate viral movement, eventually accelerating the spread of virus throughout the host system^24–26^. Using *Tobacco mosaic virus* (TMV) MP as a specific ligand, researchers found that a calreticulin protein binds with TMV-MP^27^. Calreticulin is a key retention factor in maintaining proper protein folding in the ER^28^. In addition to its ER distribution, calreticulin also targets PD^29^. Overexpression of calreticulin formed a filamentous ER network and severely blocked intercellular movement of TMV^27^, supporting the notion that the structure of ER is crucial for PD function.

To simulate the impact of virus infection, a pumpkin *(Cucurbita maxima)* phloem-expressed protein, Phloem Protein16 (Cm-PP16), which is an endogenous protein equivalent to virus MPs, was used as a bait to coimmunoprecipitate tobacco (*Nicotiana tabacum*) plasmodesmal-enriched cell wall preparations. Several PD-associated proteins have been identified, such as plasmodesmal germin-like protein1 (PDGLP1). The association of PDGLP1 with secretory vesicles and their subsequent delivery to PD are mediated by the ER–Golgi secretory pathway. In addition, PDGLP1 interacts with numerous PD-associated proteins, including actin, PDBG, and PDGLP2, establishing a protein complex to modulate PD plasticity and functionality^30^. Furthermore, Kraner et al. employed a PD-localized movement protein (MP17) encoded by Potato leafroll virus as an indicator to create a forward genetic screen in an attempt to identify host factors that regulate PD biogenesis and development. The results from this study, surprisingly, identified CTL1, alternatively referred to as Choline transporter-like 1 (CHER1), whose mutation altered the binding pattern of MP17 to PD^31^. A mutant of the *CHER1* gene had no obvious effect on the generation of primary PD; the biogenesis of secondary PD was, in contrast, drastically impaired. Interestingly, the maturation of PD from simple to complex was almost abolished in the *cher1* mutant, indicating the crucial role of CHER1 during PD maturation^31^. The localization of CHER1 to the TGN compartment and early endosomes validates the possible involvement of CHER1 in regulating the proper migration of vesicles from Golgi to PD. To provide evidence for this hypothesis, the *cher1* mutant was employed for in-depth fractionation and quantitative high-resolution mass spectrometry analysis. Corresponding results from filtering and comparative analyses have identified 61 PD-associated proteins that are significantly downregulated in the *cher1* mutant. These downregulated proteins also include a number of previously described PD-localized proteins and several uncharacterized proteins, including calcium/lipid-binding proteins as well as heavy metal transporters^32^. These findings provide a valuable data set that could be used to explore potential regulatory mechanisms associated with PD formation. Although available evidence supports the importance of the ER–Golgi secretory pathway during PD formation, the underlying mechanism remains an enigma.

### ER–PM contact and ER–desmotubule

PD channels are formed by the apposition of two membrane systems: the PM and the ER membrane. These two close apposition membrane systems contain highly specialized domains that likely play roles in PD biogenesis, reshaping, and functionality. Electron tomography has provided unprecedented insights into the 3D ultrastructure of PD and sheds light on the physical plasticity of the ER–PM interface^4^. The ER–PM interface undergoes extensive remodeling, which may promote the transition of PD from type I (tightened contact between the ER and the PM) to type II (with obvious intermembrane gaps), indicating that ER–PM contacts control PD maturation and define the differential functions of PD channels^4^. In yeast and mammals, ER–PM contacts function as special docking sites for lipid transfer and interorganelle communication^33,34^. In plant cells, ER–PM contacts particularly control cell-to-cell connectivity through PD^35,36^. Synaptotagmin A (SYTA), which is located at ER–PM contacts, functions as a tethering protein to maintain ER morphology and stabilizes the formation of ER–PM contacts^37^. In contrast to the reticulate morphology of cortical ER in the WT, the ER in *syta-1* mutants is unstructured and fails to support the typical ER polygonal structure. Disrupted ER in *syta-1* mutants resulted in the blockage of PD permeability and impeded the cell-to-cell movement of viruses^37,38^. To further investigate the precise distribution of Synaptotagmin, Ishikawa et al.^39^ employed super-resolution confocal live imaging microscopy and successfully demonstrated that *Arabidopsis* Synaptotagmin 1 (SYT1) specifically localizes to the ER–PM boundary. Additional time-lapse imaging shows a frequent distribution of SYT1 at the edges of the ER sheets, which transforms into immobile ER tubules over time^39^. However, the positioning and morphology of PD in the *syta-1* mutant is still unknown. SYTA, the best-studied protein in the SYT1 family, which is well known for its involvement in membrane trafficking, is also involved in the initiation of exocytosis and calcium (Ca^2+^) sensing^40^. Therefore, these associated functions hint at the possible contributions of ER–PM contacts to Ca^2+^ homeostasis. A decrease in intracellular Ca^2+^ concentration caused by chemical treatment triggers pronounced fragmentation and segregation in the ER–PM contacts^39^, supporting the important role of Ca^2+^ in the maintenance of ER shape and PD architecture.

Recent research findings show that the formation of desmotubules plays an instrumental role in shaping ER–PM contacts and modulates the proper curvature of ER and membrane structures^36^. The reticulon (RTNLB) family of ER-tubulating proteins that are abundant at PD facilitates the constriction of ER membranes to enhance the formation of desmotubules. Owing to the dimerization/oligomerization of RTNLB proteins, ER tubulation and desmotubule membrane constriction are enhanced to generate a unique curvature of the membrane at PD^41,42^. This type of membrane curvature mediates the selective localization of lipid-associated protein sorting at the liquid-ordered membrane phase^43^, further creating a proper lipid environment at the ER–PM contacts^44^. Therefore, RTNLB-mediated ER–desmotubule formation at PD favors the localization of lipid-anchored proteins at PD and easy exchanges of lipids^7^. The well-established lipid exchange prevailing at the ER–PM contacts enables rapid membrane remodeling at PD and thus facilitates the dynamic opening and closure of PD.

### Cytoskeleton

The cytoskeletal system, which includes actin and microtubules, provides the fundamental structural framework for shaping cell morphology. The establishment of ER–PM contacts has been implicated in the intracellular movement and positioning of organelles, which particularly requires cytoskeleton regulation^45,46^. Actin and associated motor proteins, such as myosins, within PD components serve as scaffolds to connect desmotubules and PM^47^. Blockage of actin polymerization by Latrunculin B and the overexpression of myosin tails that impair the normal function of endogenous myosins effectively disrupted PD permeability^48^. Therefore, actin and myosins are assumed to be essential components in the control of PD functionality^48^.

Interestingly, studies have shown that Kinesin G (KinG), a motor protein that moves along microtubule filaments, interacts with SHR protein, which is known to shuttle from cell to neighboring cell via PD^49^. KinG localizes predominantly to the microtubule and overlaps with actin. Moreover, KinG only interacts with the mobile form of SHR, and cell-to-cell movement of SHR is compromised in the *kinG* mutant^50^. These observations support the involvement of actin and microtubules in the assembly and trafficking of cellular components through PD.

In addition to KinG, the actin-binding Networked (NET) 1 A protein, which binds actin and is located at PD, functions as an adaptor protein to connect actin with PD^51^. NET3C, which is a homolog of NET1A in *Arabidopsis*, is associated with actin and localizes exclusively to the ER–PM contacts^52^. The available evidence indicates that the association of NET3C with ER–PM contacts relies on the formation of a protein complex through the dimerization/oligomerization of NET3C and VAMP-associated-protein 27 (VAP27) (a plant homolog of yeast Scs2 ER–PM contact site protein) in an actin- and microtubule-dependent manner^52,53^. Given the crucial contribution of the cytoskeleton to the establishment of ER–PM contacts, researchers have proposed that the actin-microtubule network promotes the stabilization of the NET3C-VAP27 protein complex at the ER–PM contacts^52^ and is thus a key factor in PD biogenesis.

Recent investigations have revealed that another actin-binding protein, called Formin 2, is responsible for actin targeting to PD^54^. PD permeability increases in the *Arabidopsis formin 2* mutant; meanwhile, the involvement of Formin 2 in the regulation of PD depends on the interaction between Formin 2 and actin^54^.

Altogether, the intimate association of actin, myosin and microtubule with PD further supports the hypothesis that the cytoskeleton is an integral component of PD. However, contrary to the conclusion of the above studies, Nicolas et al.^4^ showed that treatment of roots with F-actin polymerization inhibitors had no adverse effect on PD structure. Thus, the exact contributions of the cytoskeleton to PD functionality is still unclear.

### Specific PD membrane lipid composition

PD are membrane-lined pores, indicating the importance of membranes in defining the functionality of the PD. The membrane constituents comprise lipid rafts enriched with phospholipids, sphingolipids, and sterols^55^. These lipid components partition the membrane into 10–200 nm nanodomains that are collectively referred to lipid rafts^55^. These lipid components are significantly enriched in PD membranes compared with the neighboring PM, which facilitate the establishment and maintenance of a unique signaling platform for the communication of PD-located proteins^7^. A well-characterized lipid nanodomain-binding protein, called REM, has been reported to modulate PD-dependent virus movement by creating specialized subcompartmentation of membranes^56^. Interestingly, REM is also detectable at PD and interacts with viral MPs to impair their ability to increase the PD aperture^57–59^. Very recent studies have demonstrated that rice stripe virus interferes with S-acylation of *Nicotiana benthamiana* (Nb) REM1 and thus interferes with its autophagic degradation process^60^; *Arabidopsis* REM1.3’s phosphor-status determined its membrane nanodomain organization and was crucial for virus movement via PD^61^. These results support the important roles of REM proteins during virus infection, possibly by modulating PD membrane organization.

Some PD-located proteins, such as PDBGs and PDCBs, belong to the group of GPI-anchored proteins, which are also associated with the sphingolipid and sterol-enriched membrane nanodomains^62–65^. Disruption in the normal PD localization patterns of PDCB1 and PDBG2 in response to the sterol synthesis inhibitor treament^7^ underscores the crucial role of lipid rafts in the maintenance of the PD membrane system. Interestingly, the GPI motif has been identified as a primary PD sorting signal to conduct GPI-anchored proteins residing at the PD^63^. Thus, the GPI motif represents a powerful molecular tool for studying the progression of signaling events at PD.

The preferential enrichment of lipid rafts at PD and the existence of a close relationship between PD-localized proteins and lipid rafts provide a scenario in which the PD membrane establishes a dynamic lipid raft-abundant platform to foster the partitioning of membrane nanodomains under varying circumstances. Operationally, the organization pattern of these nanodomains facilitates the compartmentalization or partitioning of the PD membrane-associated proteins. Changes in the lipid phase are vital to control membrane flexibility and consequently influence the plastic opening and closure of PD.

### Cell walls

Insights gained from plasmolysis studies showed that the PM shrinks away from the cell wall to generate hechtian strands that serve as connections between the PM and cell wall^66^. Hechtian strands are likely to correspond to PD structures^66^. Consequently, treatment with a cellulose inhibitor, which hydrolyzes cellulose from the cell wall, resulted in the collapse of the hechtian strands^66^. These observations support the possible involvement of the cell wall in the formation of PD. Incidentally, cell wall thickening coincides with transformation in PD structure under certain conditions. For instance, simple PD structures are often present in thin and newly formed cell walls, whereas complex PD structures with multiple channels and a branched morphology are usually associated with thicker and older cell walls^4^. Cell wall remodeling enzymes that cause cell wall modifications may result in changes in PD structure^67^. However, the direct influence of cell walls on PD architecture remains elusive. A recent study reported the localization of a cell wall-loosening protein, named *Nicotiana benthamiana* α-expansin (NbEXPA1), to PD in response to virus infection. The recruitment of NbEXPA1 to PD allows it to interact with viral RNA polymerase, which subsequently promotes viral movement. Although the transcript levels of *NbEXPA1* were downregulated during viral infection, silencing of *NbEXPA1* impaired virus movement through PD^68^. Therefore, coordinating PD permeability contributes to defense and may involve the tightening/loosening of the cell wall^68^.

### Chloroplast

The GFP-fused expression of a phloem-specific Sucrose-H^+^ Symporter 2 promoter (pSUC2-GFP) passively transports GFP protein through the PD into the surrounding tissues; thus, pSUC2-GFP transgenic plants are usually used as a marker to dissect PD permeability^12^. Utilizing pSUC2-GFP seeds, a mutagenesis library was established to further screen PD conductivity-regulated mutants^69^. The results showed that the *gat1* mutant has a severe restriction on the transport of GFP proteins out of the root phloem^69^. *GAT1* encodes a plastidial thioredoxin gene (TRX-m3) and is expressed in non-green plastids. The *gat1* mutant caused promotion of a branched or occluded PD structure and the abnormal accumulation of callose and hydrogen peroxide^69^. Although the GAT1 protein is not specifically associated with PD, its influence on redox homeostasis seems to be indirectly involved in the regulation of PD architecture.

A mutant of chloroplast-resident DEAH-type RNA helicase, Increased Size Exclusion Limit2 (*ise2*), showed a higher incidence of PD branching^70,71^, similar to *gat1*. ISE2 protein is expressed in the cytoplasm, often close to chloroplasts^70^, again indicating a relationship between chloroplasts and PD. Given the characteristics of ISE2, which is a chloroplast-associated RNA helicase and influences plastidial RNA splicing^72,73^, the impaired PD structure in *ise2* indicates the possible involvement of chloroplast RNA in the regulation of PD architecture.

Chloroplast and PD organelles are spatially distant, and how chloroplast signals are transmitted to the PD is unresolved. In photosynthetic tissues, chloroplasts are close to mitochondria, peroxisomes, and the ER. The photosynthesizing chloroplast functions as a source of redox and reactive oxygen species (ROS)^74^. Bobik et al.^76^ proposed that ROS constitute a signaling bridge connecting the chloroplasts to the PD^75^. Accordingly, low concentrations of H_2_O_2_ that decrease ROS levels increase PD permeability, while higher concentrations of H_2_O_2_ restrict PD conductivity, supporting the essential role of ROS in the control of PD conductivity.

In addition to the abovementioned endogenous components involved in the modulation of PD structure and conductivity (Fig. 1), PD permeability is also sensitive to environmental stimuli such as phytohormones (auxin, gibberellic acid, salicylic acid), chitin, and calcium (summarized in a previous review^77^). Thus far, the regulation of PD permeability by environmental signals mostly relies on the homeostasis of callose, and the need for other regulatory mechanisms is still an open question.

## Implications of PD-mediated symplastic signaling studies for horticultural research

Studies of the model plant *Arabidopsis* have helped to elucidate the genetic mechanisms underlying the dynamic regulation of PD opening and closure and have attracted extensive attention. Insights gained from studies conducted on this model plant have shown that PD-related biotechnological engineering could significantly enhance research efforts aimed at improving desirable agronomics traits in a wide range of horticultural crops.

### Grafting

Grafted plants often have a massive advantage over their parents. This technique is widely employed in the horticulture industry, which involves combining the root system from one variety onto the shoot from another to generate a chimeric organism^78,79^. The shoot section of one plant used in the union is termed the scion, and the root system of a different plant is termed the rootstock^80^. Successful grafting only requires vascular reconnection across the graft junction. During the vascular reconnection process, PD are primarily formed de novo in the phloem sieve element, facilitating the transportation of photosynthetic assimilates from scion to the rootstock. Thus, de novo PD biogenesis is crucial for determining the success of grafting^81^. The functional number of PD and the efficiency of symplastic transport across the graft junction are obviously different in compatible grafts compared with incompatible grafts^82^. Incompatible graft junctions contain diverse structures and compositions of PD between the two combined plants and thus usually cause a failure of PD connection. For example, when an apricot cultivar with a poor transport system grafts on a plum cultivar with an efficient transport system, PD-mediated symplastic communication is reduced considerably compared with the performance of plum in homografts^82^.

**Fig. 2:**
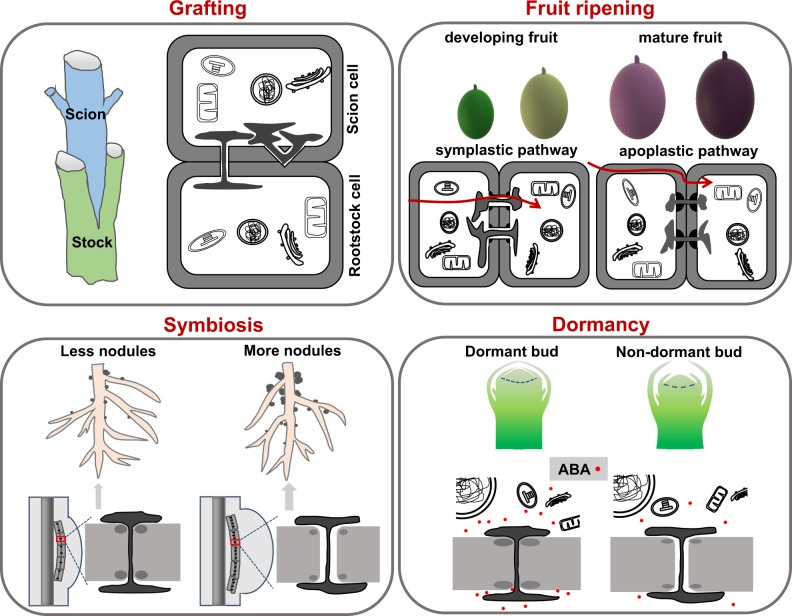
PD morphology in horticultural plants. Appropriate manipulation of PD conductivity can improve horticultural plant traits, including the processes in grafting, fruit ripening/quality, symbiosis, and dormancy period. Dynamic PD opening-closure (shown in the cartoon) corresponds to individual plant growth status or development stage: (1) During the scion-stock grafting process, de novo PD formation determines the success of grafting and the efficiency of symplastic transport across the graft junction. (2) During the fruit ripening process, the pathway switched from symplastic (corresponding to the developing fruit) to apoplastic (corresponding to the mature fruit), which provides an essential development checkpoint to indicate fruit ripening. (3) During the symbiosis process, nodule initials require the successful establishment of PD-mediated symplastic communications. Excess callose deposition impairs PD conductivity and reduces nodule number, and a lower callose level promotes PD permeability and increases nodule number. (4) During the dormancy process, a high ABA level enhances callose deposition at PD, resulting in PD closure and bud dormancy; in contrast, a low ABA level decreases callose level and promotes PD reopening, releasing the dormant bud from dormancy

Based on the grafting system, Molnar et al.^83^ employed mutants that impede small RNA (siRNA) biogenesis in either source or recipient tissues and found that these 24-nucleotide mobile siRNAs can move through PD across the graft junction. Because of the mobile ability of siRNA, the genome of the recipient cells undergoes direct modifications via the donor plant^83^. To date, PD-mediated transport of silencing signals from cell-to cell has been utilized in the cultivation of horticultural plants, as shown by siRNA-mediated gene silencing in intact potato rootstock grafts with a siRNA produced tobacco scion^84^. Thus, through the usage of grafting technology, PD-mediated RNA movement appears to be an excellent tool to generate plants that could be of horticultural or scientific interest (Fig. 2).

### Fruit quality

Fresh fruits taste sweet owing to the high concentration of sugar, mostly sucrose. Sucrose is transferred by the phloem from the photoassimilating aerial tissues to the distant root system or fruit for metabolism and storage. The transportation of sucrose occurs through two routes: the PD-mediated symplastic pathway and sucrose transporter-dependent apoplastic pathway^85^. The transport of sucrose through the phloem into the fruits demonstrates the transfer of sucrose from sieve elements (companion cells) to the sink (parenchyma cells) for storage^86^. Different plants use different mechanisms to transport sucrose. For example, apples use an apoplastic pathway during fruit development^87^. In grape berries, the symplastic pathway operates in the early stages and then shifts to the apoplastic pathway in the later stages of fruit development^88^. Researchers compared Chinese cultivated jujube fruit that contains high sugar concentrations with wild-type sour jujube containing less sugar. Here, Zhang et al.^89^ demonstrated that PD-mediated symplastic transportation is only present in cultivated jujube but not in sour jujube, suggesting the pivotal role of PD-mediated symplastic sucrose transportation for the improvement of fruit quality.

Through the detection of symplastic transportation and TEM observations of PD structure in different fruit stages of the grape berry, PD-mediated symplastic transport is abolished at or just before the onset of the ripening stage, corresponding to a shift from the symplastic to apoplastic transportation pathway^88^. In the oil palm, the switch from symplastic to apoplastic transportation relies on the morphological characteristics of the fruit’s primary abscission zone, which harbors many PD during the early stages of the fruit but a drastically reduced PD number during fruit ripening^90^. This type of PD distribution and morphological divergence are correlated with cell wall remodeling during different developmental stages of fruit development^90^. Hence, the switch of sugar transportation modes provides a structural checkpoint to indicate fruit ripening^88,91^ (Fig. 2).

Although symplastic and apoplastic pathways for sugar transportation are strictly separated, cantaloupe fruit, which relies mainly on the PD-mediated symplastic sugar transport pathway, also incorporates an apoplastic mode in response to viral infection^92^. Therefore, the manipulation of PD-mediated symplastic pathways for sugar transportation is crucial for plant nutrient reallocation during plant development and responses to pathogen challenges.

### Symbiosis

Legume plants adopt a unique strategy to enhance nitrogen acquisition by generating root nodules, also called plant–microbe symbiosis. This symbiotic process is highly correlated with carbon partitioning and sugar transport, involving a series of cell morphological changes^93^. During nodule initiation, the differentiation of root pericycle cells and de novo formation of nodule primordia correspond to the establishment of a PD continuum that precedes cell division events^94^. By TEM observations, PD channels were shown to be established de novo in the cell walls of the stele/pericycle, pericycle/endodermis, and endodermis/inner cortex interfaces, which are associated with the activation of nodule initial cells during *Medicago* nodule formation^94^. The transgenic expression of TMV-MP in *Medicago*, which is known to increase PD permeability, significantly increased nodule number^94^. A recent study showed that rhizobia inoculation promotes the reduction of callose levels in nodule initial cells and thus increases the symplastic connectivity between phloem and nodule initial cells^18^. Downregulation of callose levels by overexpressing the *Medicago* BG2 enzyme significantly enhanced nodule number, and upregulation of callose content by ectopically overexpressing *cals3m* decreased nodule number^18^. Thus, the rearrangement of the symplastic network in the nodule primordium results in an increase of phloem unloading of shoot-derived carbohydrates and thus promotes nodule initiation.

Cells must consume energy to maintain the sugar transport activity from the apoplast. Compared with apoplastic sugar transport, the symplastic pathway occurs passively and shows less energy consumption^95^. In *Casuarina glauca* nodules, PD channels in the infected cells are much more abundant than in the uninfected cells^95^, indicating that nodules largely rely on this less energy-demanding symplastic pathway to supply sugars to the infected cells. Compared with young infected cells, PD density is much lower and branched PD is absent in mature infected cells, which might be explained by the degradation of PD correlated with cell lignification of mature nodules^95^. Therefore, the establishment of PD-mediated symplastic communications is a critical element for the successful establishment of the rhizobia–legume association (Fig. 2).

### Dormancy

The shoot apex of overwintering plants temporarily ceases their physical activity and shifts into a bud that is dormant and freezing-tolerant. Once the temperature is suitable for plant growth, the dormant bud gradually recovers by breaking dormancy, called release. Dormancy is an important period as the plant cells tend to eliminate the associations with environmental conditions to conserve energy. Breakage of bud dormancy involves restoration of nutrient and signal transportation. Recent studies have shown that dormancy and release events are also directly correlated with the dynamic opening and closure of the PD in different plant species. In maize, chilling that causes dormancy leads to a severe architectural change in PD in leaf mesophyll cells, which show strong sphincter swelling and constriction of the cytoplasmic sleeves of PD^96^. Bilska and Sowinski compared chilling-sensitive maize with a chilling-tolerant line and found that calreticulin accumulated particularly in the neck region of PD in the chilling-sensitive line. Given its calcium buffering activity, calreticulin participate in PD gating probably via modulation of calcium concentration^29^. In birch and Norway spruce, short photoperiods promote dormancy and stimulate the accumulation of callose at PD^97,98^. Deposition of callose results in PD closure and symplastic isolation of cells on apical meristem cells^97,98^. In contrast, activation of PDBGs triggers the removal of callose and PD reopening for initiation of symplastic communication to release the dormant bud from dormancy^97,98^.

ABA acts as a positive regulator to stimulate PD closure^99^. In hybrid aspen trees, ABA signaling stimulates dormancy and promotes PD closure in WT but not in ABA-insensitive mutant *abi1*. Interestingly, many PD-associated proteins are differentially expressed in the WT and *abi1*, implying that PD functionality directly correlates with ABA signaling^99^. Researchers grafted scions of WT and *abi1* mutants with a short photoperiod (to induce PD closure and dormancy) onto rootstocks of FLOWERING LOCUS T1-expressing plants that promote dormancy release, and new leaves emerged only in *abi1* scions but not in WT scions^99^. Hence, ABA-mediated PD closure plays a crucial role in supporting the survival of plants under changing environmental conditions (Fig. 2). Manipulation of plastic PD opening-closure by ABA is an applicable method in horticulture research to fine-tune the plant dormancy period.

In summary, PD closure arrests plant growth and protects the plant against sudden changes in the environment. Hence, dynamic regulation of PD opening-closure can be an approach to prevent precocious activation of growth, which is crucial for perennial survival and longevity^98^.

## Outlook

PD-mediated symplastic transport acts as a unique cell–cell communication pathway in multicellular plants, which is required for their coordinated growth and development. PD channels dynamically adjust their structure and aperture to facilitate intercellular transportation of various micro- and macromolecules. The plasticity of the PD architecture is maintained by essential intracellular organelles, including the ER, vesicles, the cytoskeleton, the PM, the cell wall, and chloroplasts. Callose homeostasis has been proposed as one of the most important factors that modulates the permeability of PD during organ development and the response to biotic/abiotic stresses (summarized in ref. ^11^). However, the regulatory mechanisms of the temporal and spatial regulation of callose deposition at PD remain largely unknown. Although ER–PM contacts are likely responsible for the biogenesis of simple PD, the operational mechanisms of branched PD formation are still unclear. Microtubules and actin constitute a fundamental cytoskeleton system for all aspects of organelle movement/formation, but their contribution to PD plasticity is still under debate. In regard to the PD structure, current instrumentation developments will help to solve open questions, especially new advances in super‐resolution imaging and targeted genome editing, which show promise for elucidating the structure and functionality of PD.

A long-term goal of agricultural/horticultural research is to enhance crop yield and improve the nutritional quality of edible organs. The pivotal role of PD in plant defense responses is acknowledged, and this physical defense strategy can be a promising approach to block the intercellular spread of pathogens^19,100^. Furthermore, regulation of PD conductivity provides an unexploited potential biotechnological technique to improve sugar transportation, to enhance the success of grafting and protect plants against adverse environmental conditions. Therefore, PD biotechnological engineering represents a promising tool for breeding horticultural crops with desired traits.
